# Supplementary figures and images for: Induction of HIV-1-specific antibody-mediated effector functions by native-like envelope trimers in humans
Source: PLoS Pathog. 2025 Oct 21;21(10):e1013614. doi: 10.1371/journal.ppat.1013614 (PMC12551954; doi:10.1371/journal.ppat.1013614)

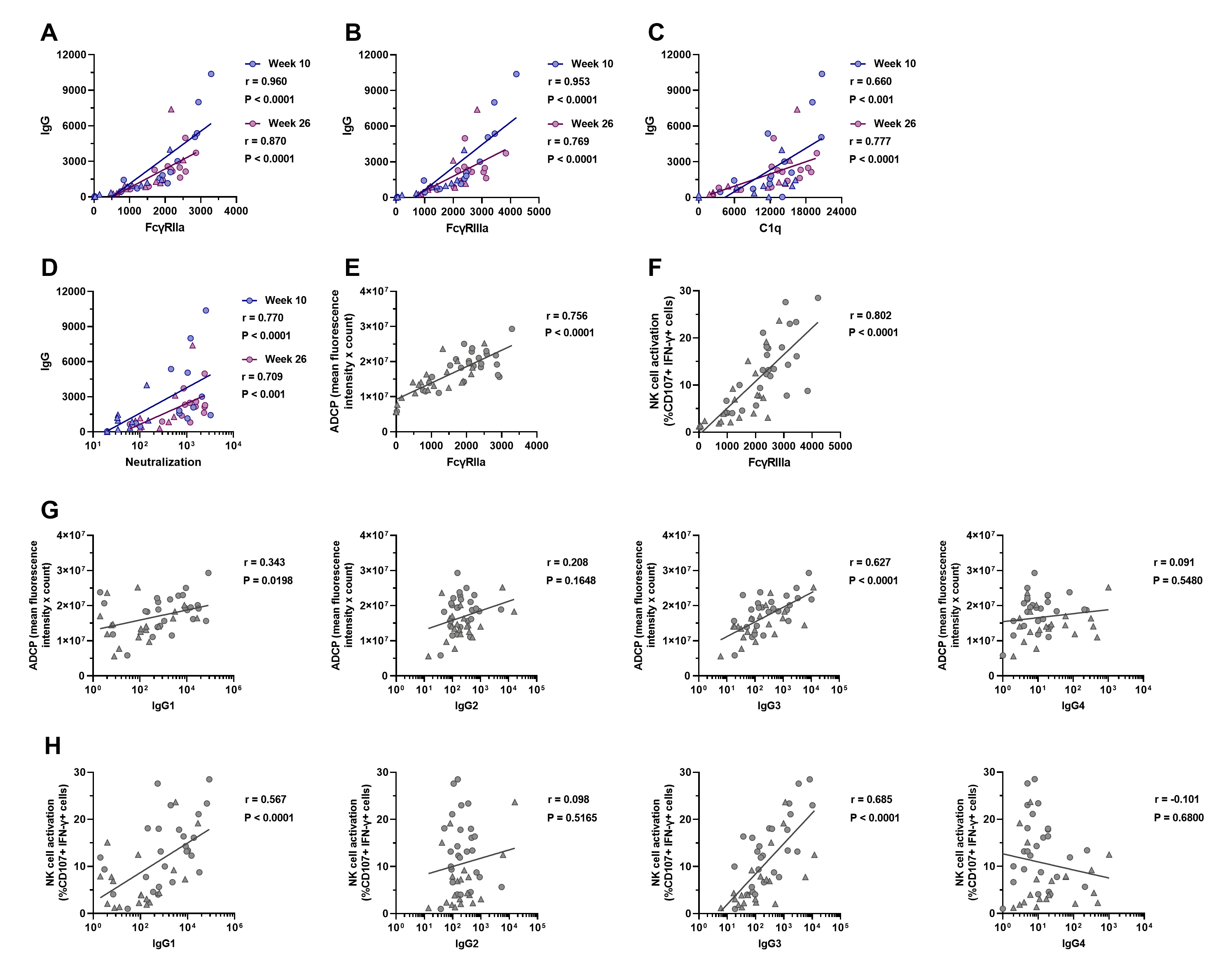

Supplement: S1 Fig — Spearman correlations between ConM-specific IgG levels and interaction of ConM-specific antibodies with (A) FcγRIIa, (B) FcγRIIIa and (C) C1q. (D) Spearman correlations between ConM-specific IgG levels and half-maximal infective dilution (ID50) neutralization titer against ConM pseudovirus. (E) Spearman correlations between interaction of ConM-specific antibodies with human FcγRIIa and antibody dependent cellular phagocytosis (ADCP) of ConM SOSIP.v7 conjugated beads by THP-1 cells. The ADCP score is composed of the mean fluorescence intensity multiplied by the count of internalized beads. (F) Spearman correlations between interaction of ConM-specific antibodies with human FcγRIIIa and activation of NK cells derived from healthy donor PBMCs by ConM-specific serum antibodies. The NK cell activation is presented as the percentage of cells expressing CD107 and IFN-γ. Spearman rho correlation coefficients (r) and P-values are indicated separately for the serum collected at week 10 and week 26. All IgG, FcγRIIa, FcγRIIIa and C1q data shown are median fluoresence intensity (MFI) measured by Luminex assay. Female participants are shown as circles and males as triangles. (TIF) [file ppat.1013614.s001.tif]

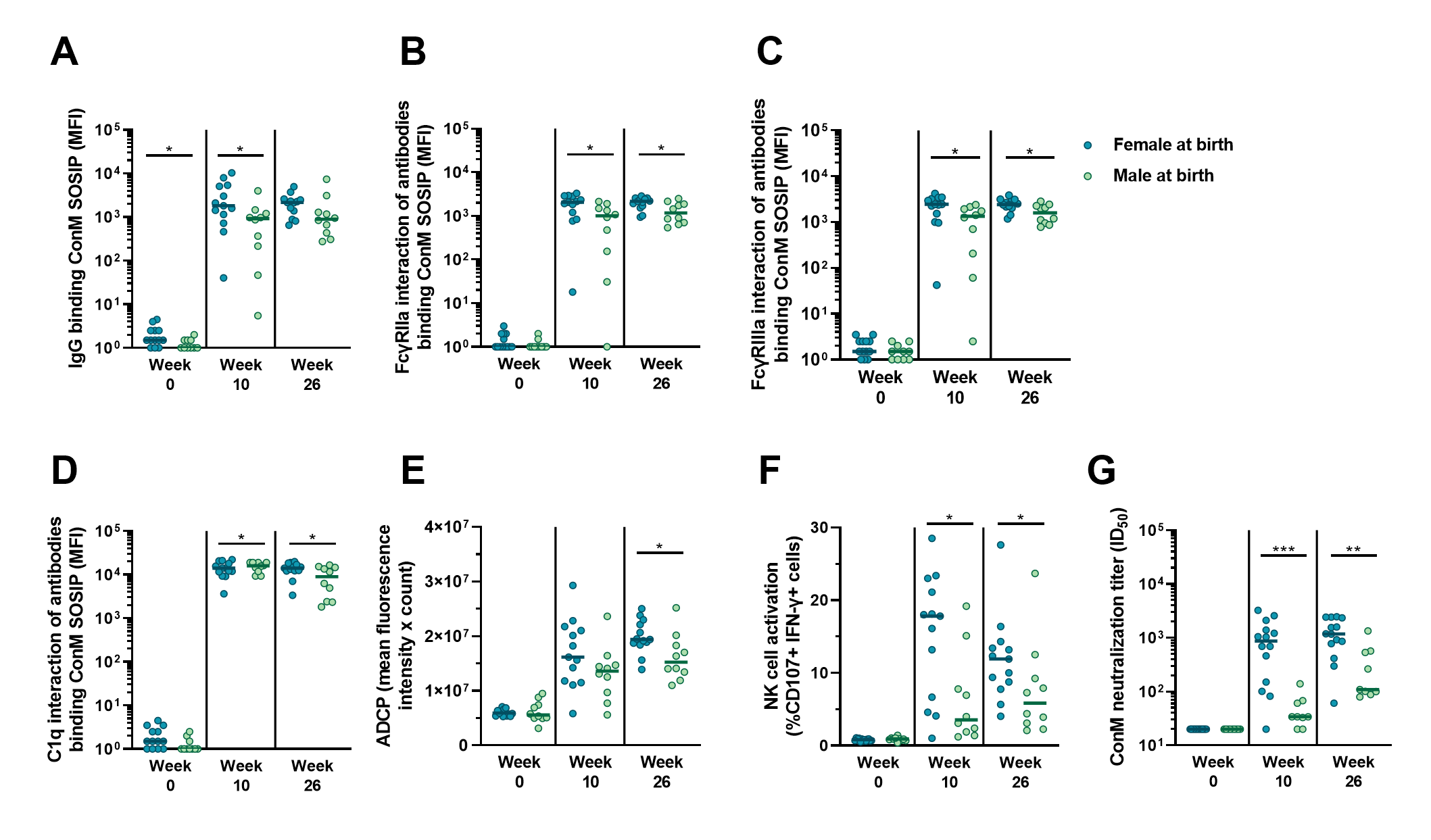

Supplement: S2 Fig — (A) Male and female participants were compared at baseline, after two vaccinations and after three vaccinations for IgG levels, (B) interaction of ConM-specific antibodies with human FcγRIIa, (C) human FcγRIIIa and (D) human C1q. All measurements were performed by Luminex immunoassay and presented as blank-corrected median fluoresence intensity (MFI). (E) Antibody dependent cellular phagocytosis (ADCP) of ConM SOSIP.v7 conjugated beads by THP-1 cells mediated by serum antibodies compared between male and female clinical trial participants. The ADCP score is composed of the mean fluorescence intensity multiplied by the count of internalized beads. (F) NK cell activation by ConM-specific serum antibodies, presented as the percentage of cells expressing CD107 and IFN-γ. (G) ID50 neutralization titer against ConM pseudovirus compared between clinical trial participants with a full or fractional third dose. Groups were compared at each time point with a Mann-Whitney U test. * = P < 0.05, ** = P < 0.01, *** = P < 0.001. (TIF) [file ppat.1013614.s002.tif]

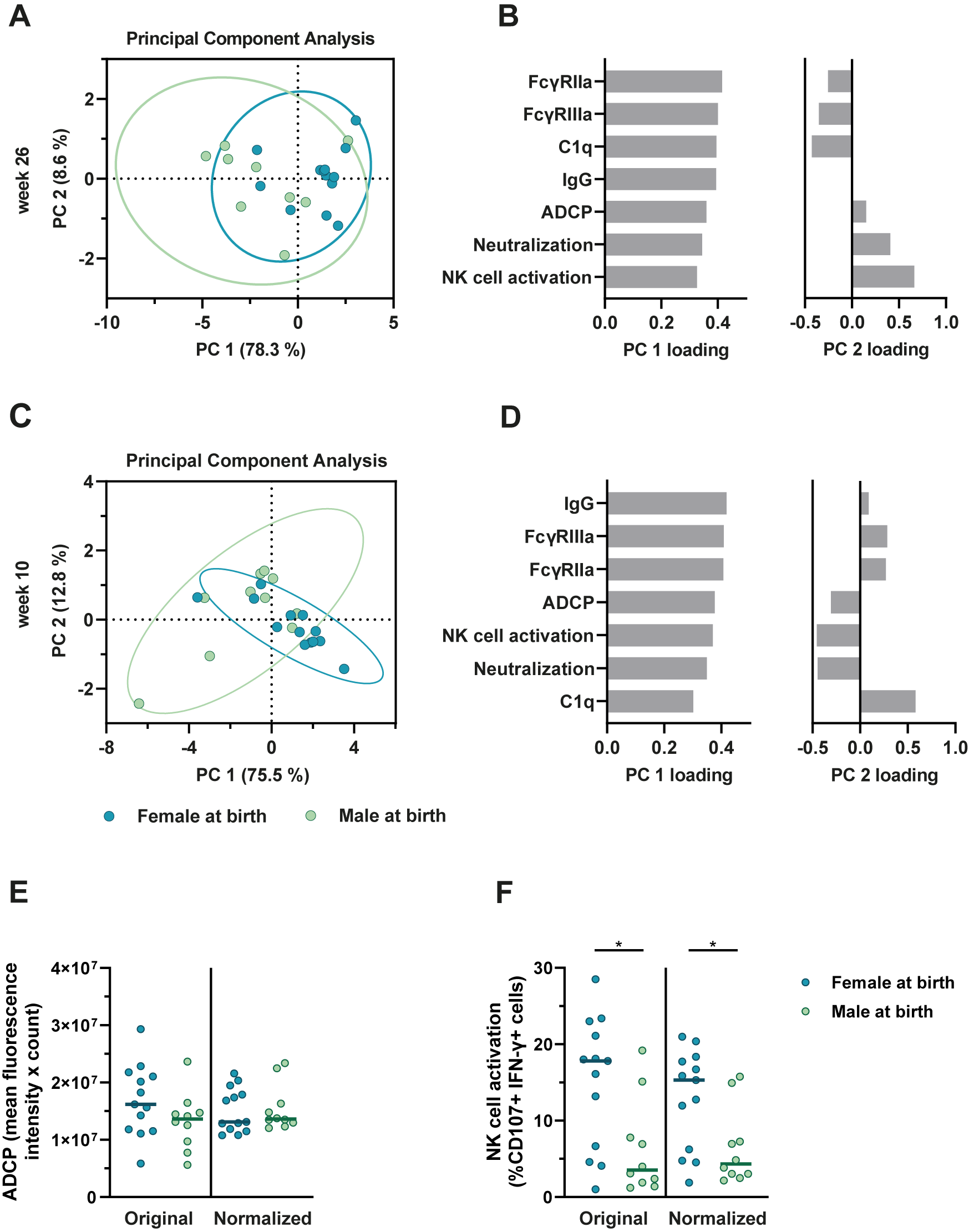

Supplement: S3 Fig — (A) Principal component analysis (PCA) over all data presented in the graphs in Fig 2 to identify the features that differ the most between female and male participants. Axes of the graph indicate the percentage of data variance explained by the first two principal components (PC). (B) Loading bar charts explaining the contribution of the different measurements to the separation of the groups on PC 1 and PC 2. (C) PCA for serum responses after two vaccinations (week 10) to identify the features that differ most between male and female participants. (D) Loading bar charts explaining the contribution of the different ConM-specific measurements to the separation of the groups on PC 1 and PC 2. (E) ADCP and (F) NK cell activation responses at week 10, normalized by IgG levels. Week 10 IgG levels were log-transformed and then a normalization factor was calculated as the fold-change in log IgG titers compared to the average log IgG titer. Results for ADCP and NK cell activation where multiplied by this factor to obtain the normalized values. * = P < 0.05. (TIF) [file ppat.1013614.s003.tif]

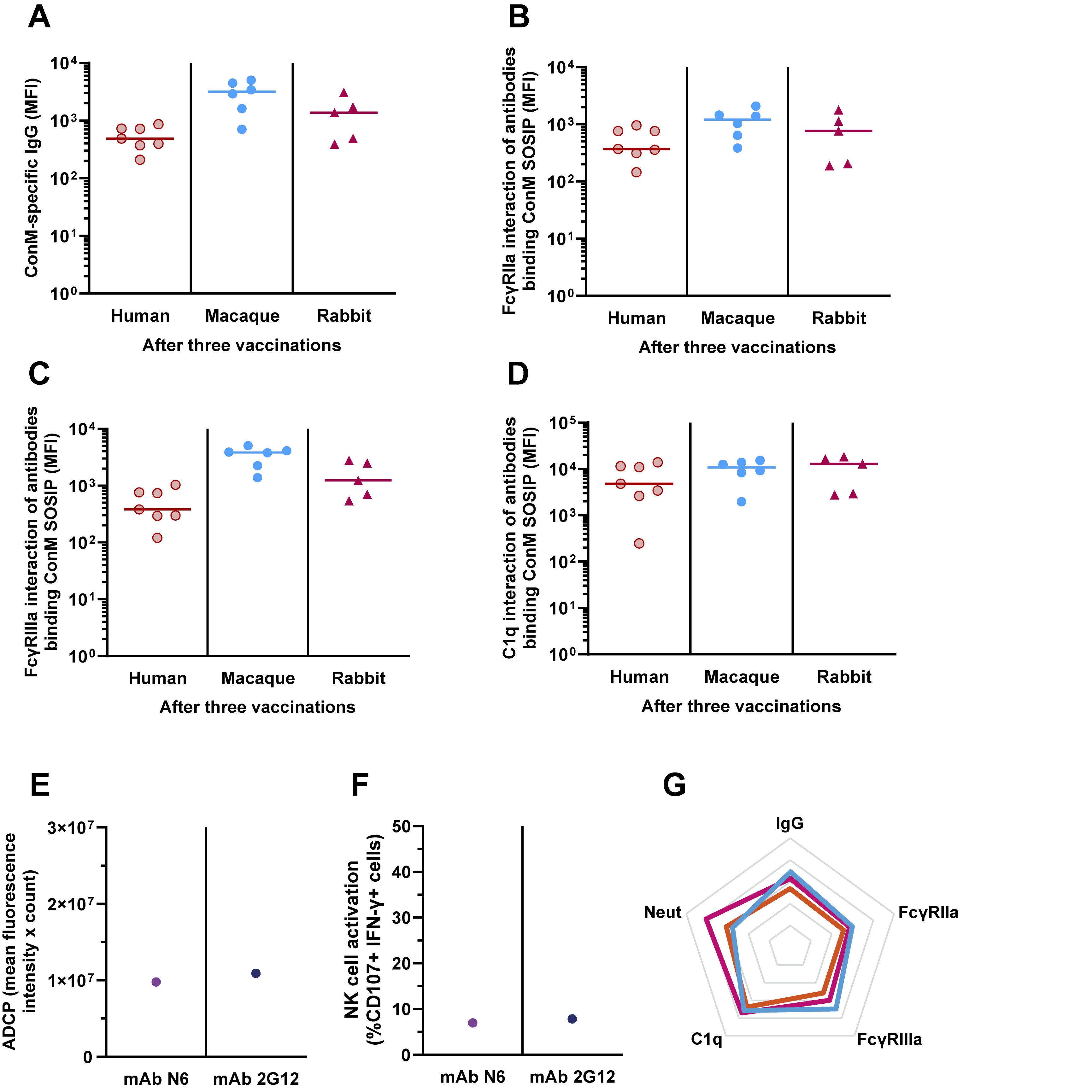

Supplement: S4 Fig — (A) ConM-specific IgG levels expressed as median fluorescence intensity (MFI) measured by Luminex assay after three vaccinations (week 26 for macaques and the human study, week 22 for rabbits). Species-specific secondary antibodies were used. (B) Interaction of ConM-specific antibodies with FcγRIIa expressed as MFI measured by Luminex assay after three vaccinations. Human FcγRIIa was used for the human and rabbit study while cynomolgus macaque FcγRIIa was used for the macaque study. (C) Interaction of ConM-specific antibodies with FcγRIIIa expressed as MFI measured by Luminex assay after three vaccinationsHuman FcγRIIIa was used for the human and rabbit study while cynomolgus macaque FcγRIIIa was used for the macaque study. (D) Interaction of ConM-specific antibodies with C1q expressed as MFI measured by Luminex assay after three vaccinations. Human C1q was used for all three studies. Sera of each species were diluted equally to facilitate these comparisons. Since the data comprised different species, the results were assessed qualitatively and without statistical analysis. Monoclonal antibodies with known effector function activity included in the ADCP and NK cell activation assays as controls. The monoclonal antibodies were tested at a concentration of 1 µg/mL and the sera shown in Fig 3 were tested at a dilution of 1:500. (G) Overlay of the three spider plots shown in Fig 3. (TIF) [file ppat.1013614.s004.tif]

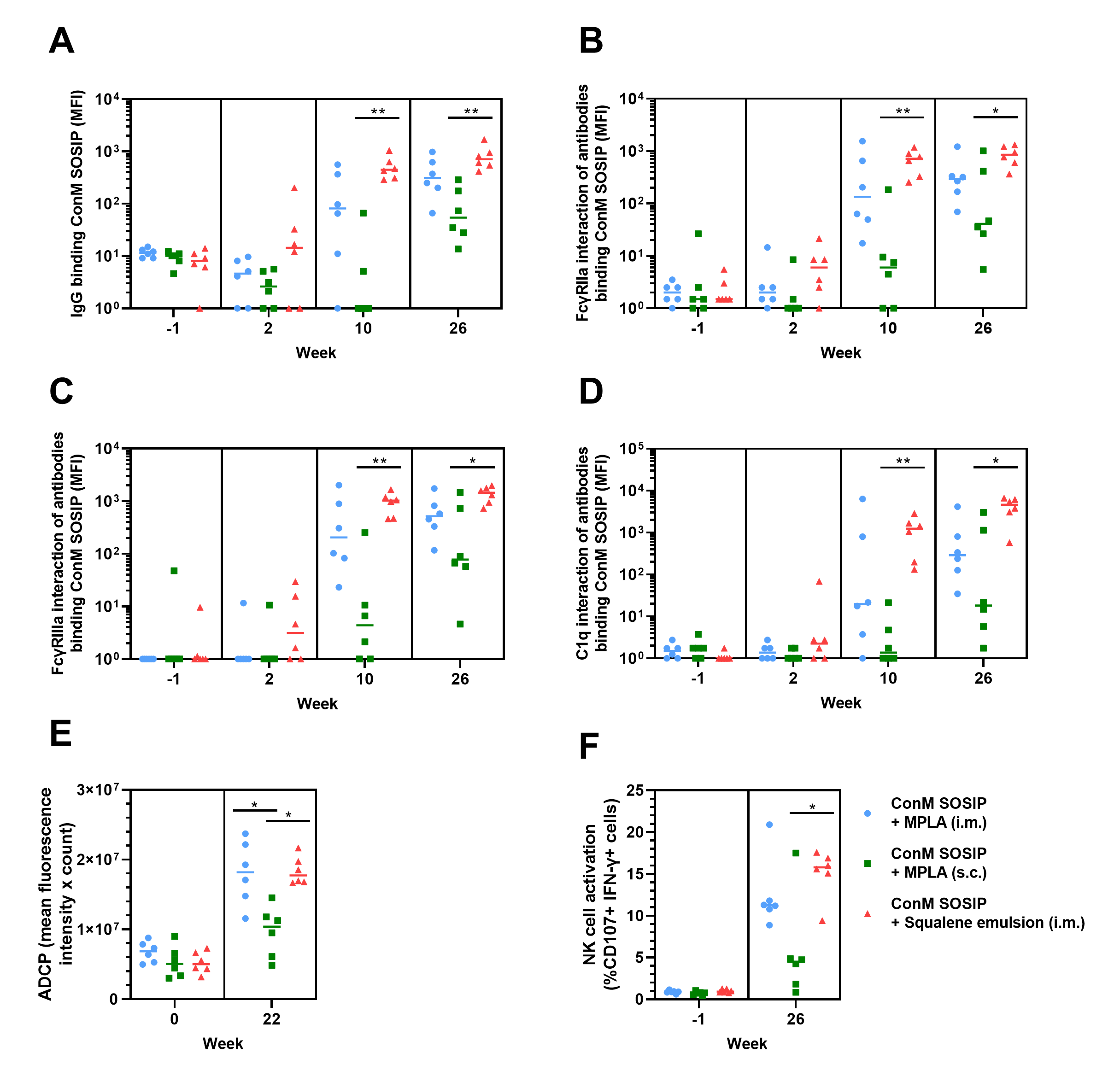

Supplement: S5 Fig — (A) ConM-specific IgG levels in serum before vaccination (week -1), after one vaccination (week 2), after two vaccinations (week 10) and after three vaccinations (week 26). (B) Interaction of ConM-specific antibodies with cynomolgus macaque FcγRIIa. (C) Interaction of ConM-specific antibodies with cynomolgus macaque FcγRIIIa. (D) Interaction of ConM-specific antibodies with human C1q. Data for panels A-D was measured by Luminex immunoassay and is presented as blank-corrected median fluoresence intensity (MFI). (E) Antibody dependent cellular phagocytosis (ADCP) of ConM SOSIP.v7 conjugated beads by THP-1 cells mediated by serum antibodies at baseline (week -1) and after three vaccinations (week 26). The ADCP score is composed of the mean fluorescence intensity multiplied by the count of internalized beads. (F) NK cell activation by ConM-specific serum antibodies before vaccination (week -1) and after three vaccinations (week 26), presented as the percentage of cells expressing CD107 and IFN-γ. At each timepoint, all three groups were compared with each other using a Kruskal-Wallis test followed by a Dunn’s multiple comparison test. * = P < 0.05, ** = P < 0.01. (TIF) [file ppat.1013614.s005.tif]

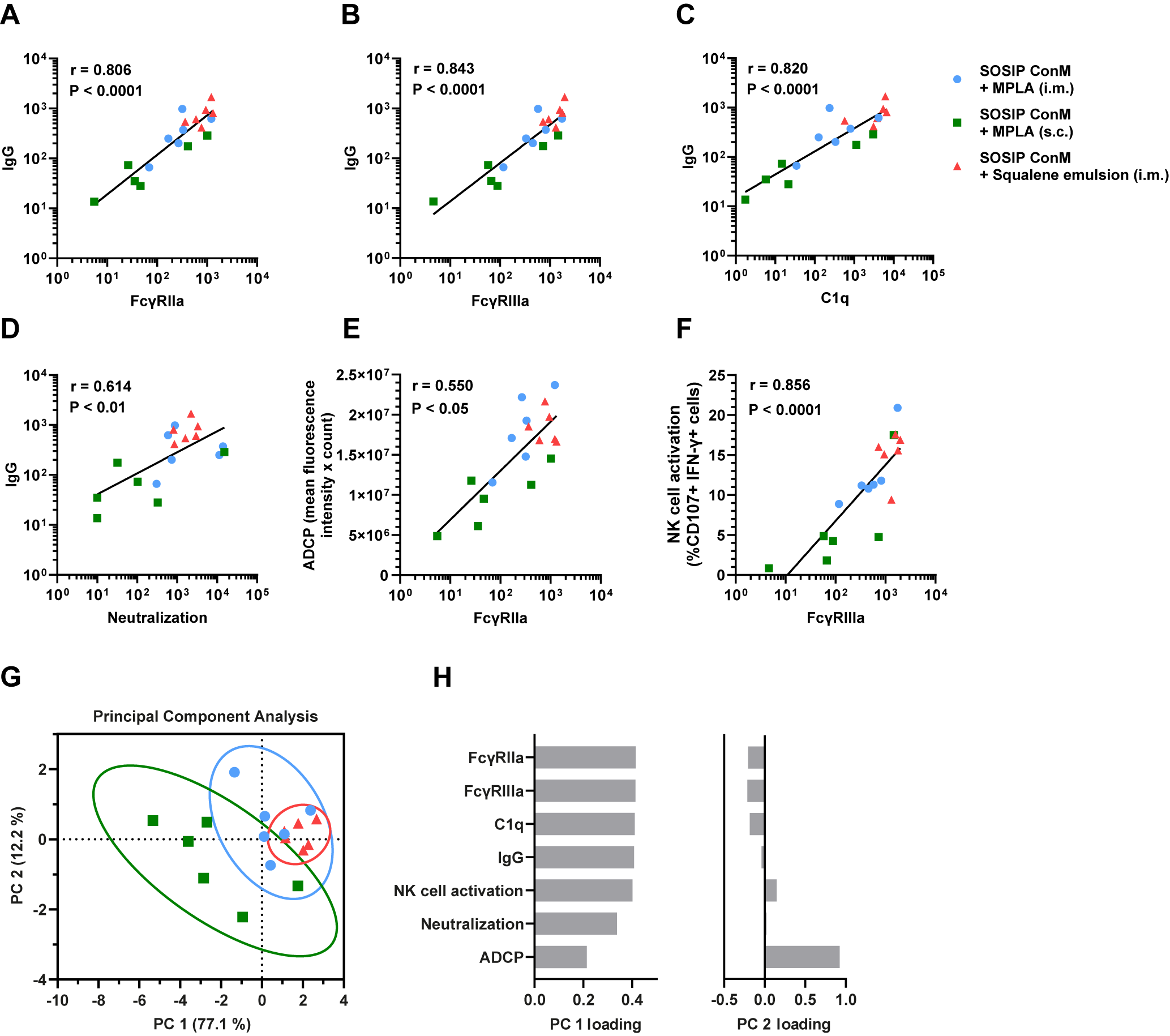

Supplement: S6 Fig — Spearman correlations between ConM-specific IgG levels and interaction of ConM-specific antibodies with (A) cynomolgus macaque FcγRIIa, (B) cynomolgus macaque FcγRIIIa and (C) human C1q. (D) Spearman correlations between ConM-specific IgG levels and half-maximal infective dilution (ID50) neutralization titer against ConM pseudovirus. (E) Spearman correlations between interaction of ConM-specific antibodies with cynomolgus macaque FcγRIIa and antibody dependent cellular phagocytosis (ADCP) of ConM SOSIP.v7 conjugated beads by THP-1 cells mediated by serum antibodies. The ADCP score is composed of the mean fluorescence intensity multiplied by the count of internalized beads. (F) Spearman correlations between interaction of ConM-specific antibodies with cynomolgus macaque FcγRIIIa and NK cell activation by ConM-specific serum antibodies, presented as the percentage of cells expressing CD107 and IFN-γ. Spearman rho (r) correlation coefficients and P-values are indicated on each graph. All IgG, FcγRIIa, FcγRIIIa and C1q results shown are median fluoresence intensity (MFI) measured by Luminex assay on serum collected two weeks after the third vaccination (week 26). (G) Principal component analysis (PCA) over all data presented in the graphs of Fig 4 to identify features that differ the most between experimental groups. Axes of the graph indicate the percentage of data variance explained by the first two principal components (PC). (H) Loading bar charts explaining the contribution of the different measurements to the separation of the groups on PC 1 and PC 2. (TIF) [file ppat.1013614.s006.tif]

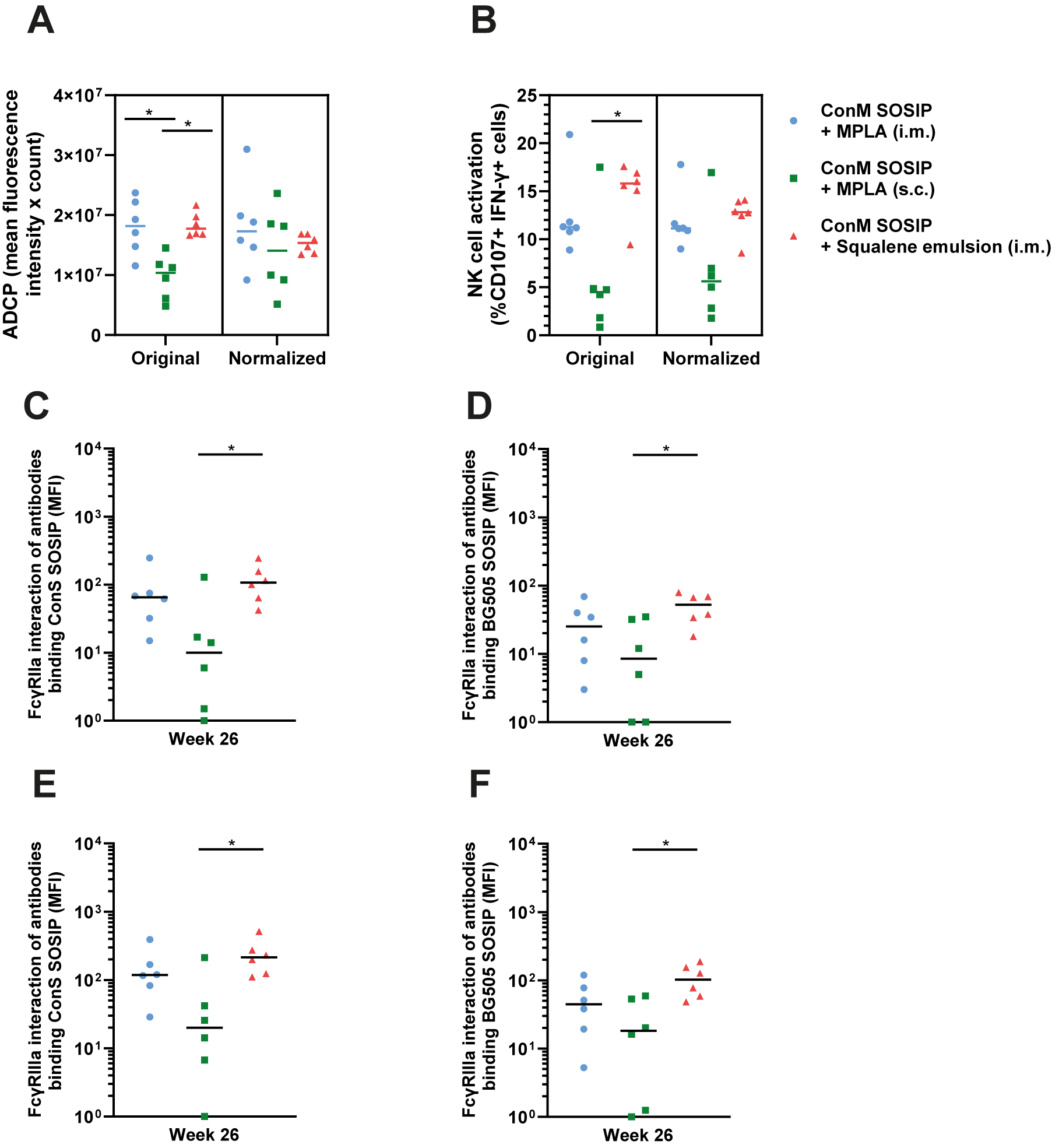

Supplement: S7 Fig — (A) ADCP and (B) NK cell activation responses at week 26, normalized by IgG levels. Week 26 IgG levels were log-transformed and then a normalization factor was calculated as the fold-change in log IgG titers compared to the average log IgG titer. Results for ADCP and NK cell activation where multiplied by this factor to obtain the normalized values. (C) The intraction of antibodies binding the heterologous antigens ConS SOSIP and (D) clade A BG505 SOSIP with FcγRIIa. (E) The intraction of antibodies binding the heterologous antigens ConS SOSIP and (F) clade A BG505 SOSIP with FcγRIIIa. Data for panels C-F was measured by Luminex immunoassay and is presented as blank-corrected median fluoresence intensity (MFI). In each graph, all three groups were compared with each other using a Kruskal-Wallis test followed by a Dunn’s multiple comparison test. * = P < 0.05. (TIF) [file ppat.1013614.s007.tif]

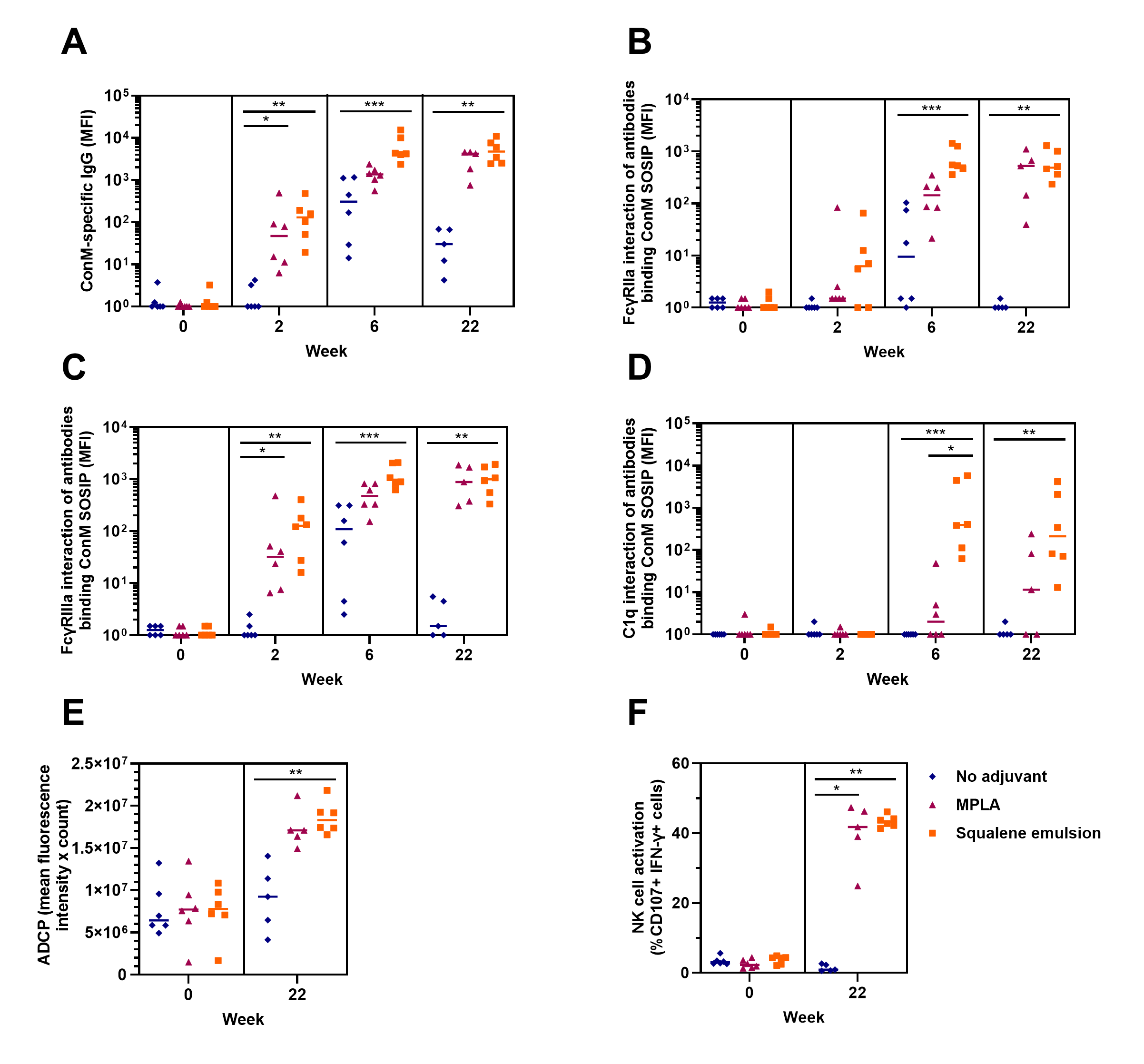

Supplement: S8 Fig — (A) ConM-specific IgG levels in serum before vaccination (week 0), after one vaccination (week 2), after two vaccinations (week 6) and after three vaccinations (week 22). (B) Interaction of ConM-specific antibodies with human FcγRIIa at the same time points. (C) Interaction of ConM-specific antibodies with human FcγRIIIa. (D) Interaction of ConM-specific antibodies with human C1q. Data for panels A-D was measured by Luminex immunoassay and presented as blank-corrected median fluorescence intensity (MFI). (E) Antibody dependent cellular phagocytosis (ADCP) of ConM SOSIP.v7 conjugated beads by THP-1 cells mediated by serum antibodies at baseline (week 0) and after three vaccinations (week 22). The ADCP score is composed of the mean fluorescence intensity multiplied by the count of internalized beads. (F) NK cell activation by ConM-specific serum antibodies before vaccination (week 0) and after three vaccinations (week 22), presented as the percentage of cells expressing CD107 and IFN-γ. At each timepoint, all three groups were compared with each other using a Kruskal-Wallis test followed by a Dunn’s multiple comparison test. * = P < 0.05, ** = P < 0.01, *** = P < 0.001. (TIF) [file ppat.1013614.s008.tif]

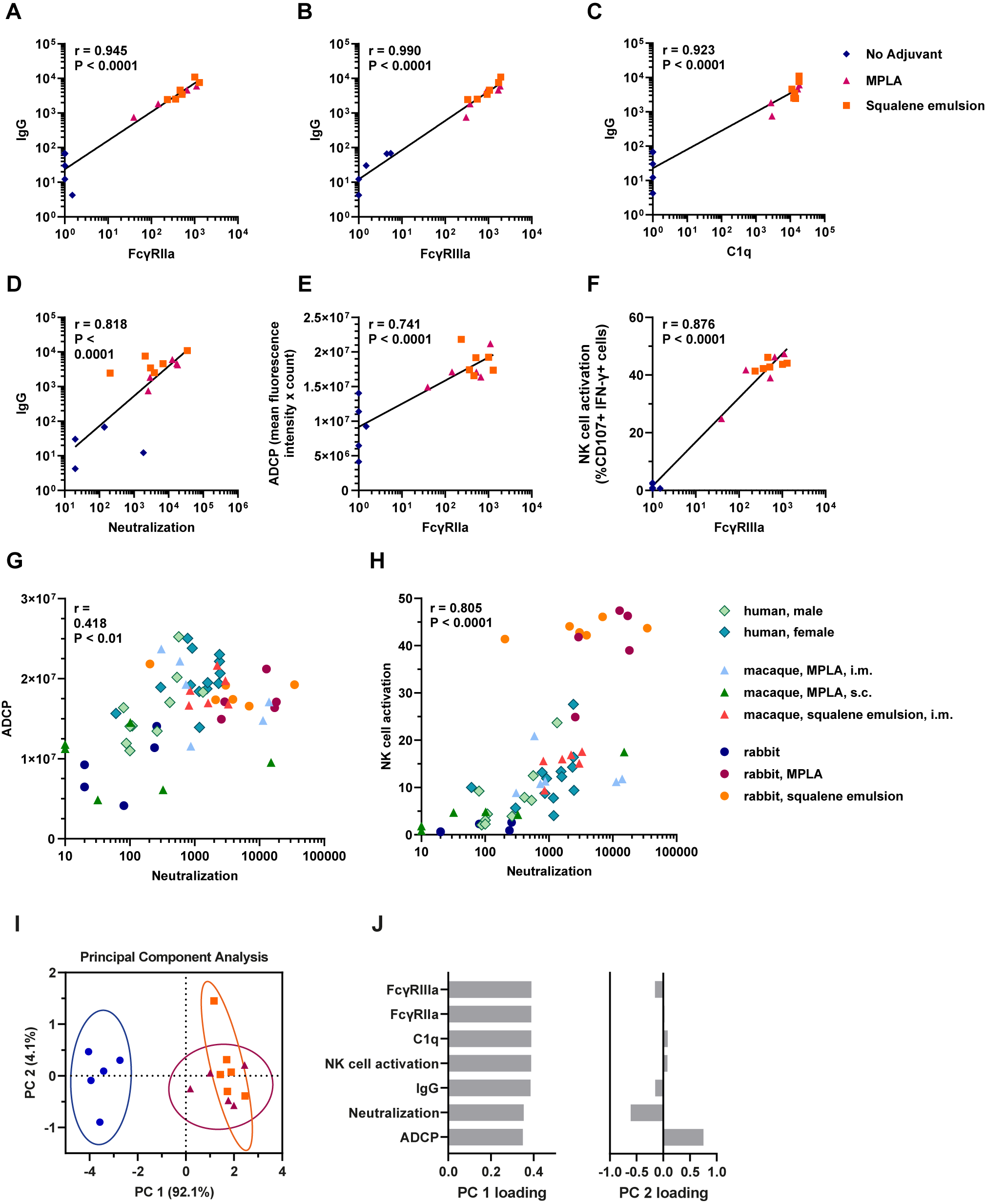

Supplement: S9 Fig — Spearman correlations between ConM-specific IgG levels and interaction of ConM-specific antibodies with (A) human FcγRIIa, (B) human FcγRIIIa and (C) human C1q. (D) Spearman correlations between ConM-specific IgG levels and half-maximal infective dilution (ID50) neutralization titer against ConM pseudovirus. (E) Spearman correlations between interaction of ConM-specific antibodies with human FcγRIIa and antibody dependent cellular phagocytosis (ADCP) of ConM SOSIP.v7 conjugated beads by THP-1 cells mediated by serum antibodies. The ADCP score is composed of the mean fluorescence intensity multiplied by the count of internalized beads. (F) Spearman correlations between interaction of ConM-specific antibodies with human FcγRIIIa and NK cell activation by ConM-specific serum antibodies, presented as the percentage of cells expressing CD107 and IFN-γ. All IgG, FcγRIIa, FcγRIIIa and C1q results shown are median fluoresence intensity (MFI) measured by Luminex assay on serum collected two weeks after the third vacciation (week 22). (G) Correlations analysis combining ADCP scores and neutralization titers at two weeks after the third vaccination for all three species (human, macaque and rabbit). (H) Correlations analysis combining the percentage of activated NK cells and neutralization titers at two weeks after the third vaccination for all three species (human, macaque and rabbit). Only human subjects that received the full dose regimen (N = 13) were incuded in the analysis for panels G and H. Spearman rho (r) correlation coefficients and P-values are indicated on each graph. (I) Principal component analysis (PCA) over all data presented in the graphs of Fig 5 to identify the features that differ the most between experimental groups. Axes of the graph indicate the percentage of data variance explained by the first two principal components (PC). (J) Loading bar charts explaining the contribution of the different measurements to the separation of the groups on PC 1 and PC 2. [file ppat.1013614.s009.tif]
