# Supplementary material for: Induction of HIV-1-specific antibody-mediated effector functions by native-like envelope trimers in humans
Source: PLoS Pathog. 2025 Oct 21;21(10):e1013614. doi: 10.1371/journal.ppat.1013614 (PMC12551954; doi:10.1371/journal.ppat.1013614)
Supplement: Attachment — Submitted filename: Response to reviewers.docx [file ppat.1013614.s012.docx]

**Response to reviewers**

For the manuscript: “*Induction of HIV-1-specific antibody-mediated effector functions by native-like envelope trimers in humans”* by Marloes Grobben *et al.*

Reviewer’s comments are shown in black, our responses are shown in *italic blue*.
All line numbers refer to the line numbers in the document with track changes.

*We thank all reviewers for their thorough feedback on our manuscript and hope we have satisfactorily addressed their concerns.*

**Reviewers' Comments:**

**Part II – Major Issues: Key Experiments Required for Acceptance**

**Reviewer #2:**

1. The detection of IgG titer was species specific and the macaque and human Fc receptors were species specific. It is not clear why the rabbit samples were screened using human receptors?

*The reviewer makes an astute observation. To our knowledge, no methods with rabbit-derived receptors and immune proteins have been developed yet. This may be in part because human reagents are also compatible with rabbit sera and have been used before as referenced in the discussion in lines 534-535. We have now specifically mentioned that a rabbit-specific method does not exist yet in the discussion in lines 535-536.*

In addition the functional assays performed (ADCC and ADCP) made use of human cell lines, diluting the applicability of comparisons made across species. As such at least an attempt should be made to use primary macaque cells in at least one of the assays to show that these results are comparable.

*We have not been able to use macaque or rabbit cells in our experiments because we do not have any experience with those cells. Establishing the required methodology would be complex and we therefore considered it beyond the scope of this study. While it would be nice to complement this work with species-matched effector cells in future studies, we feel that the fact that we see activation of human effector cells by rabbit and macaque sera was already worth describing. We now also discuss this in the manuscript (lines 547-549).*

In addition, the conclusions drawn here do not match the data – 3E for example shows a 2-fold increase in ADCC of rabbit sera compared to all others.  *We agree that the conclusions were a bit strong especially considering this finding for rabbits. We have formulated the conclusion in the results more carefully (line 302) and also adapted this in the discussion (line 515-518) and the final conclusions (line 587).*

Also it would be helpful to overlay the spider plots.

*We have now included an overlay of the spiderplots from Figure 3B in supplementary figure 4.*

2. The authors should normalise for IgG titer (this may exclude C1q binding)
*With the method we used, it is unfortunately very difficult to normalize the data based on IgG titer. This is because the data is not linearly distributed across the total range. Normalizing would then skew the data unfairly. On the one hand, there are datapoints in our dataset that are on the assay limit of detection (MFI value of 1), and on the other hand, although none of our data is saturated, we do have some data which is not linearly distributed in the upper range of the assay. While the Luminex assay we used has quite a large range where signal can be measured linearly, for some analyses there was too large variation between samples and groups to measure all the data in the linear range. Taking these considerations into account, we are left with limited possibilities of obtaining linearly distributed data for both the IgG titer and the functionality to normalize the data. We have now added analyses where we were able to normalise for IgG titer in supplementary figure 3 and 7.*

3. Are there antibody controls that the authors can include in these analyses to show that these are indeed potent Fc responses?

*We included antibody controls in the experiments previously shown to mediate effector functions as well as virus neutralization (antibodies N6 and 2G12). There was little difference between the two mAbs in both assays. For ADCP, the functionality of our 1:500 sera of all species was generally 1-2x higher than that of 1 ug/mL mAb. For NK cell activation, our data is more varied, but encompasses about 1x to 6x higher signal compared to 1 ug/mL mAb. This data has now been included in Supplementary figure 4, however we do not feel that this gives much added biological relevance to describe Fc responses as effective or not. There is no known threshold for an effective response, and combination between different Fc functions and neutralization will determine the eventual effectiveness. This has now been clarified in the discussion (lines 589-591).*

**Reviewer #3:**

The lack of testing against a more relevant target - HIV virus or infected cells is a substantial limitation. Addressing this would substantially strengthen the conclusions.

*We selected bead and plate-based assays for ADCP and NK cell activation to maximize the robustness of the data and comparison across studies. We considered using assays with virus or infected cells as they are arguably biologically more relevant. However, this also introduces additional variation and generates less robust data compared to the assays we used for the study. At the same time, the assays we used do correlate well with outcomes from assays using infected cells, as we have previously demonstrated in* [*https://doi.org/10.1038/s42003-024-06659-8*](https://doi.org/10.1038/s42003-024-06659-8)*. We now also mention this in the manuscript (lines 728-729).*

At minimum it would be helpful to evaluate breadth against a different trimer as this particular antigen was designed to address the problem of HIV env diversity.

*The breadth of immune responses elicited by this antigen have been described before in* [*https://doi.org/10.1038/s41467-019-10262-5*](https://doi.org/10.1038/s41467-019-10262-5) *and are also discussed in our submitted manuscript reporting the primary data of the clinical trial (referenced as Reiss et al., submitted, throughout our manuscript). Regarding Fc functions, we have observed outcomes very similar to those of IgG responses, with detectable but lower IgG reactivity with heterologous antigens such as ConS and BG505 Env. This data has now been included in supplementary figure 7.*

Limitations of the study should be addressed in the discussion.
*We have added discussion to more specifically address the limitations of our study (lines 495-496, 535-536, 547-549 and 566-567)*

**Part III – Minor Issues: Editorial and Data Presentation Modifications**

**Reviewer #1:**

This manuscript covers several different aspects relating to effector functions. I found the introduction slightly disjointed when these different aspects were introduced.
*We appreciate this comment and have made some changes to the introduction to improve the structure (lines 81-160).*

The NK activation assay shows some donors with a low response despite high FcyRIIIa binding of all samples. Does this not suggest that the functional assay is a better measure of the ADCC activity or that the binding assay is over saturated? What is a meaningful level of ADCC activity for protection?
*Comparing NK activation and FcyRIIIa binding is comparing two different types of data, with quite a different type of distribution. The correlation between these two datasets in supplementary figure 1F, shows that the data do correspond well and indicates that the binding assay was not saturated. There is no known threshold for a protective response, and a combination between different Fc functions and neutralization will likely determine the eventual effectiveness of a vaccine-elicited immune response. This has now been clarified in the discussion (lines 589-591).*

Were the experiments in figure 3 all done with human Fcg receptors? Wouldn’t you expect the different species to react differently? I can see this information in the figure legend but might be helpful to have in the text as well.
*Indeed this information is included in the figure legend, but for completeness we have now also added it in the text on lines 290-291.*

For panel 3D, it says that sera was diluted equally to facilitate the comparisons but should these be noramlised to the level of trimer binding antibodies to better compare?
*Normalization by trimer binding antibodies negatively impacts the quality of the data since our data is not completely linear. We feel that this does not allow for quantitative comparison. Therefore, we chose to compare Fc binding with IgG trimer binding in figure 3, by normalizing all data the same way to facilitate qualitative comparison. In addition, we were able to obtain some linearly distributed data for both IgG trimer binding and antibody functionality in a few cases. We have now added additional analyses where we were able to normalize for IgG trimer binding in supplementary figure 3 and 7. Also see our response to reviewer #2.*

Are the differences observed in 3D due to binding to the rabbit IgG FcgR or different epitopes being targeted that facilitate effector functions differently?
*Indeed figure 3D contains some striking differences. The antigen used for immunization of the three different species was identical, with similar immunization schedules and adjuvants. Therefore, different epitope targeting is unlikely, although not impossible. The species differences could potentially influence the ratio of targeted epitopes and thereby influence the level of effector functions. This has been added to the discussion (lines 543-544). While the immunization schedule for the rabbits was slightly condensed, we think this is unlikely to cause a higher functional response. We discussed that these differences could be related to the notably lower levels of fucosylation of rabbit antibodies compared to human antibodies, and we have now edited the discussion to make this hypothesis more clear (lines 544-547).*

In figure 4, the authors show that the different immunization schedules give different absolute amounts of trimer reactive antibodies. This seems to reflect the results for the remaining panels suggesting that the different vaccine protocols are impacting the overall magnitude of the response rather than any specifics about the quality of the effector function response. What would happen if the amount of Trimer-reactive antibody was normalised in the experiment to then determine if there was a change in the effector function activity? Has this been tested?
*The type of data we collected is not suitable to normalize for trimer-reactive antibodies because it is not completely linear. For now, we chose to qualitatively compare the different antibody functions to the levels of trimer-reactive antibodies (figure 3B) and we also did principal component analyses to try and delineate the subtle difference observed in our data (sup figs 3, 6 and 9). However, we have now also added additional analyses where we were able to normalize for IgG trimer binding in supplementary figure 3 and 7. Also see our response to Part II reviewer #2 and #3.*

The authors frequently refer to an unpublished study Reiss et al. It would be helpful to link the changes briefly mentioned in the text on isotype of antibodies to the effector function data seen in this paper.
*Indeed, we mentioned that the female clinical trial participants had higher levels of IgG1 subclass antibodies and lower levels of IgG4 subclass antibodies. This corresponds very well to the findings observed in the current study, where female participants had higher levels of antibodies interacting with FcyRs and C1q. We have now mentioned this more clearly in the discussion (lines 490-492). Additionally, we expect that the study by Reiss et al. will be published shortly, therefore we expect readers to be able to access and read that study as well.*

**Reviewer #2:**

1. While the authors point to the association of Fc effector function with protection in other diseases, it seems a brief summary of what the current status in HIV vaccination is, is missing in the introduction
*Indeed, we see that information on what is known on the role of antibody effector functions in HIV-1 vaccination was lacking and we have now added this (lines 105-108).*

2. The introduction could benefit from further editing – several points are reiterated multiple times and could be replaced by some background that is HIV specific
*We understand this comment and have made some changes to the introduction to improve the structure and remove redundant information (lines 81-160).*

3. The authors state: NHPs also have four IgG subclasses, but they are structurally and functionally more similar to each other than the human versions[17] but fail to mention that this a major issue especially in the context of studying very polyfunctional isotypes such as IgG3.
*Indeed, this greatly complicates studying the potential functionality of antibody responses by measuring the different IgG subclasses, as has been done in several studies for human responses, including for our clinical trial participants. Because of this, we focused only on the functionality of the response and not the type of antibodies involved. To further clarify how this poses limitations to research, such as our current study, we have now also discussed this in the discussion section of the manuscript (lines 532-534).*

4. Should the rabbit and macaque Fc responses not be tested using primary cells from the animals themselves? Surely one is underestimating the “autologous” Fc functions?
*We have not been able to use macaque or rabbit cells in our experiments because we do not have any experience with those cells. Establishing the required methodology would be complex and we therefore considered it beyond the scope of this study. While it would be nice to complement this work with species-matched effector cells in future studies, we feel that the fact that we see activation of human effector cells by rabbit and macaque sera was already worth describing, it is true that the autologous responses using species-matched cells could be more potent. We now also discuss this in the manuscript (lines 547-549). See also our response to the related part II comment of reviewer #2.*

5. In the results the authors state that “No statistically significant differences were observed between the two dosage groups after the third vaccination.” And they mention that they pooled them – it should be clarified that this refers to pooling fractionated and bolus dosing.
*We understand that this was not formulated clearly enough, and we have now modified the text to clarify that we did not differentiate between the groups with and without fractional dosing for our analyses (lines 184-185).*

Are there differences between these in Fc functions? It’s not clear whether they mean there were no differences for Nab responses etc.
*Indeed we meant that there were no differences found between the normal and fractional dosing groups in terms of IgG levels and NAb titers. Because of this, we decided not to further crowd our results section by also mentioning that there were no differences in effector functions between these groups. We have now clarified which differences were already previously observed (lines 182-184).*

6. The authors should verify that the Fc array performed has not maxed out – seems that this is the case for 1B, D and E where a very poor range of responses is noted and why no increase is seen for these conditions after the third dose. As seem to be the case for adcp and adcc – also fc effector arrays do not tell us “to what extent the elicited antibodies could mediate effector function” but rather tell us the extent to which fc receptors are bound.
*We understand the reviewers concern, that the way the data is presented in figure may appear like saturated data. This is due to the logarithmic axis and the non-linear distribution of the data. The correlation plots in Supplementary Figure 1 can confirm that none of the data is saturated. The non-linear distribution of the data makes quantitation of differences difficult, this is why we also included nonparametric statistical tests in the figures.
Why no increase in response was noted after the third dose is another question, we focused our current study on the week 26 timepoint mainly. The study by Reiss et al. which is frequently referred to in our study has looked further into the response over time. There, using different assays, there was also limited increase in the response after the third dose. We therefore do not find it surprising that there is also no increase in functional responses and we considered this out of scope for the current study since it is already discussed in the manuscript by Reiss et al.*

*In the beginning of the results, we meant to explain the relevance of measuring Fc receptor and C1q binding. We agree that this sentence was poorly formulated, and have adapted the text (lines 189-191).*

7. “Antibody functionality can be expected to be closely linked to antibody quantity.” This sentence in the context it is written is also misplaced. The Fc receptor binding affinity is linked to IgG titer as these output are measured in the same way and not accounted for when running plasma. C1q titers less so as they can be mediated by other isotypes.
*Indeed, we meant that total functionality is a sum of the functionality, here approximated through Fc receptor binding affinity, and antibody quantity. This was not well formulated. To prevent confusion, we have removed the sentence entirely (line 195).*

8. By “autologous serum neutralization” do the authors mean ConM titers?
*This was indeed not clear, we have now changed the text to call it ConM neutralization titers instead (lines 199-200).*

9. What happens to the ADCP and ADCC results if igg is normalised for? Ie if score is normalised to IgG titer? Are the authors sure that the sex differences are not because of IgG being higher in females? This difference should be accounted for. PCA in fact shows that all of the top four features are all almost equally accounting for the difference – likely because they are colinear.
*Indeed, the functionality of the antibody response is also linked to the antibody quantity, of which IgG is an important component. We do think the data suggests that not all the functional differences we observe are explained by IgG quantity, which is further supported by differences in the IgG subclass distribution found between males and females which we refer to in lines 488-490. We agree that the PCA analyses are not that clear, and have removed them from the main text and moved them to the supplement instead (supplementary figures 3, 6 and 9). We have now also added analyses where we were able to normalize for IgG trimer binding within a subset of samples in supplementary figure 3 and 7.*

10. The scale on figure 3B is confusing and should be either amended or explained
*We understand that the scale was confusing, and have adapted the scale to match the scale used in our other graphs (Figure 3B).*

11. The PCA plots drawn are not really informative and are illustrated by the differences in univariant data
*We agree that the information provided by the PCA analyses is limited, and have moved them to the supplement instead (supplementary figures 3, 6 and 9).*

12. I think more attention should be drawn to the fact that adjuvant boosting depends on the animal model tested
*We agree that there are likely differences in how adjuvants work in different animal models, we have adapted the discussion to make this more clear (lines 571-574)*

**Reviewer #3:**

Methods for neutralization assay should be included.
*We added a brief explanation on the neutralization assay used (lines 730-740).*
