# Supplementary material for: Induction of HIV-1-specific antibody-mediated effector functions by native-like envelope trimers in humans
Source: PLoS Pathog. 2025 Oct 21;21(10):e1013614. doi: 10.1371/journal.ppat.1013614 (PMC12551954; doi:10.1371/journal.ppat.1013614)
Supplement: Attachment — Submitted filename: Response to reviewers_revision2.docx [file ppat.1013614.s013.docx]

**Response to reviewers**

For the manuscript: “*Induction of HIV-1-specific antibody-mediated effector functions by native-like envelope trimers in humans”* by Marloes Grobben *et al.*

Reviewer’s comments are shown in black, our responses are shown in *italic blue*.
All line numbers refer to the line numbers in the document with track changes.

*We thank all reviewers for their thorough feedback on our manuscript and hope we have satisfactorily addressed their concerns.*

**Reviewers' Comments:**

**Reviewer #2:**

In general the authors have failed to experimentally address my concerns - while the conclusions have been tempered, I still think that the usefulness of such a study with no additional biological information is limited. The authors also state that "macaque cell usage is complex" but receptors are available commercially from these species so several high-throughput assays could be performed.

*We agree that our study contains limitations concerning how for we can approximate the true biology that occurs when immune responses are activated in vaccinated people or animals. Nevertheless, we have applied and adapted the methods that are available at this time to explore what we can learn about antibody-mediated effector functions, also in macaque and rabbit animal models. Indeed, macaque receptors are available and we did generate FcƴR reagents specific for cynomolgus macaques, which we used in our assays. This was mentioned in the results and figure legends (lines 233, 898-899 and 915) but is now also clarified in lines 262, 269, 272. Moreover, we have now additionally addressed this in the discussion (lines 406 and 409-410). Finally, we have now also added the limitations revealed during our study to the conclusion paragraph of the manuscript (lines 475-477), to further clarify that there is still a lot of work that can be done and methodology to develop to progress and mature this approach in future studies.*
